# Antibodies to SARS Coronavirus in Civets

**DOI:** 10.3201/eid1012.040520

**Published:** 2004-12

**Authors:** Changchun Tu, Gary Crameri, Xiangang Kong, Jinding Chen, Yanwei Sun, Meng Yu, Hua Xiang, Xianzhu Xia, Shengwang Liu, Tao Ren, Yedong Yu, Bryan T. Eaton, Hua Xuan, Lin-Fa Wang

**Affiliations:** * Changchun University of Agriculture and Animal Sciences, Changchun, China;; † CSIRO Livestock Industries, Geelong, Australia;; ‡ Harbin Veterinary Research Institute, Harbin, China;; § South China Agriculture University, Guangzhou; China;; ¶ Guangdong Provincial Veterinary Station of Epidemic Prevention and Supervision, Guangzhou, China

**Keywords:** SARS-CoV, antibodies, civets, dispatch

## Abstract

Using three different assays, we examined 103 serum samples collected from different civet farms and a market in China in June 2003 and January 2004. While civets on farms were largely free from SARS-CoV infection, ≈80% of the animals from one animal market in Guangzhou contained significant levels of antibody to SARS-CoV, which suggests no widespread infection among civets resident on farms, and the infection of civets in the market might be associated with trading activities under the conditions of overcrowding and mixing of various animal species.

Severe acute respiratory syndrome (SARS) first appeared in November 2002 in Guangdong Province, China (*1*). The outbreak was caused by a newly emerged virus now known as the SARS-associated coronavirus (SARS-CoV), which is believed to originate from animals. Most of the early index cases in Guangdong Province were concentrated in food handlers, and workers in live-animal markets had higher rates of antibodies to SARS-CoV than persons in other occupations (*2**,**3*). Studies have indicated that Chinese ferret-badgers (*Melogale moschata*), masked palm civets (*Paguma larvata*), and raccoon-dogs (*Nyctereutes procyonoides*) could be naturally infected by SARS-CoV or a closely related virus (*4*). Furthermore, experimental infection studies indicated that a variety of animals, including monkey, cat, ferret, mouse, and pig, are susceptible to SARS-CoV infection (*5**–**9*). These findings highlight the difficulties facing investigation into the origin of SARS-CoV.

Civets have been considered one of the most likely animals responsible for animal-to-human SARS-CoV transmission, and on this basis, more than a thousand civets in Guangdong were culled in January 2004. However, no conclusive evidence suggests that civets are the natural reservoir host of SARS-CoV or that civets in their natural habitat are infected with SARS-CoV. Lack of access to wild civets and regulatory issues involved make conducting detailed field studies of wild civets difficult, if not impossible, for the foreseeable future. Since most civets in markets are sourced from civet farms, we have conducted a preliminary serologic study on the prevalence of antibodies to SARS-CoV in civets from the market and farms.

## The Study

After detecting SARS-CoV in civets from animal markets in Shenzen in late November 2003, the Guangdong government launched a campaign to cull all civets in the province to reduce the risk of SARS-CoV transmission to humans (*10*). To study the distribution of SARS-CoV and antibodies in these culled animals, intestine tissues and serum samples were taken from 56 animals: 38 civets from four farms in different regions of Guangdong Province (10 from Zhuhai, 10 from Shanwei, 9 from Shaoguan, and 9 from Qingyuan; Figure) and 18 civets from the Xinyuan Live Animal Market in Guangzhou.

Because of time constrains and regulatory issues, selection was conducted on the basis of convenience and personal contact with groups involved in the slaughter campaign. However, we tried to select civets from farms >100 km apart in the Guangdong Province. A total of 41 civet farms were in Guangdong Province at the time of the slaughter campaign, and most had <100 animals. No biosecurity measures were used in farms or markets, and no veterinary examination or accreditation was required for civet farming or trading. All of the farms tested had obtained their original seed stock from markets.

Also included in the study were 47 civet serum samples that had been previously collected in early June 2003 from two civet farms in Luoning City of Henan Province and Changsha City of Hunan Province. The farm conditions were similar to those in Guangdong, basically small-scale farms without biosecurity or animal health safeguards.

All serum samples were inactivated at 56°C for 30 min, transferred to the Australian Animal Health Laboratory, and inactivated by gamma irradiation before analysis. Anti–SARS-CoV antibody in serum was detected by using immunofluoresence antibody assay (IFA) and quantified in a microtiter virus neutralization test (VNT). The SARS-CoV (strain HKU-39849) used in both VNT and IFA was plaque purified three times in Vero cells, and stock virus (titer 5 x 10^7^ 50% tissue culture infective dose [TCID_50_]) prepared by two low-multiplicity passes in Vero cells. In IFA, monolayers of Vero cells infected with SARS-CoV at a multiplicity of infection of 0.02 TCID_50_/cell were methanol-fixed 24 h postinfection, exposed to a range of serum dilutions, and bound antibody detected by using fluorescein isothiocyanate–conjugated protein A (Kirkegaard & Perry Laboratories, Gaithersburg, MD). Groups of samples that reacted positively in either VNT or IFA were also subjected to Western blot analysis with a recombinant SARS-CoV nucleocapsid (N) protein expressed in *Escherichia coli*. Bound antibodies were detected by using alkaline phosphatase–conjugated protein A/G (Pierce, Rockford, IL).

Intestine tissues collected from the 56 animals in January 2004 were also tested for SARS-CoV viral nucleic acid by using reverse transcription–polymerase chain reaction (RT-PCR). Total RNA was extracted from these samples by using the Trizol method (Invitrogen, Carlsbad, CA), followed by first-strand cDNA synthesis using the Superscript II RNase H reverse transcriptase (New England Biolab, Beverly, MA) and random hexamer primers. PCR amplification was conducted by using Ex Taq polymerase (TaKaRa). Three pairs of SARS-CoV–specific primers were used to amplify regions in the N gene (forward, 5´-ATGTCTGATAATGGACCCCAAT; reverse, 5´-TTATGCCTGAGTTGAATCAG), the M gene (forward, 5´-ATGGCAGACAACGGTACTATT; reverse, 5´-CTTACTGTACTAGCAAAGCAAT) and the S gene (forward, 5´-ATGTTTATTTTCTTATTATTTC; reverse, 5´-GTCGACATGCTCAGCTCCTAT), respectively.

Of 103 civet serum samples tested, 18 were positive on at least one of the three assays used, for ≈17% overall seroprevalence. However, when seroprevalence among civets from farms and the market was compared, differences were observed. For samples taken in January 2004, 14 of 18 obtained from the Xinyuan Live Animal Market in Guangzhou tested positive by all three assays (Table), for a seroprevalence of 78%. In contrast, the prevalence on each farm was <40% (4 of 10 animals from the farm in Shanwei tested positive, and no positive animals were found on the other farms); the overall prevalence on farms was 4 (≈10%) of 38. SARS-CoV antibody levels in the four animals at the farm in Shanwei, which is located ≈240 km east of Guangzhou (Figure), were lower than those from the market, and two samples positive by VNT failed to react on IFA or Western blot (Table).

**Table:** Summary of serologic analyses of civet serum samples^a^

Sample no.	Farm													Market		
	Hunan		Henan		Guangdong									Guangdong		
	Changsha		Luoning		Qiangquan		Shaoguan		Shanwei			Zhuhai		Guangzhou		
	VNT^b^	IFA^c^	VNT^b^	IFA^c^	VNT^b^	IFA^c^	VNT^b^	IFA^c^	VNT^b^	IFA^c^	WB^d^	VNT^b^	IFA^c^	VNT^b^	IFA^c^	WB^d^
1	–	–	–	–	–	–	NA	NA	10	–	–	–	–	10	+++	++++
2	–	–	–	–	–	–	–	–	10	+	+	–	–	640	++	++++
3	–	–	–	–	NA	NA	–	–	–	–	–	–	–	CONT	+++	+++
4	–	–	–	–	–	–	–	–	–	–	–	–	–	20	+++	++++
5	–	–	–	–	–	–	–	–	40	+/–	+	–	–	–	–	–
6	–	–	–	–	–	–	–	–	20	–	–	–	–	30	++	+++
7	–	–	–	–	–	–	–	–	–	–	–	–	–	–	–	–
8	–	–	–	–	–	–	–	–	–	–	–	–	–	10	++	+++
9	–	–	–	–	–	–	–	–	–	–	–	–	–	–	–	+
10	–	–	–	–	–	–	–	–	–	–	–	–	–	–	–	–
11	–	–	–	–										10	++	++++
12	–	–	–	–										NA	NA	NA
13	–	–	–	–										20	++	++++
14	–	–	–	–										30	++	++++
15	–	–	–	–										NA	NA	NA
16	–	–	–	–										10	++	++++
17	–	–	–	–										10	+++	++++
18	–	–												10	++	++++
19	–	–												240	+++	++++
20	–	–												60	+++	++++
21	–	–														
22	–	–														
23	–	–														
24	–	–														
25	–	–														
26	–	–														
27	–	–														
28	–	–														
29	–	–														
30	–	–														

^a^VNT, virus neutralization test; IFA, immunofluorescent antibody test; WB, Western blot test; NA, not assayed because of sample damage or loss during transportation or storage; CONT, contaminated samples that exhibited toxicity in VNT test. ^b^Titer of antibody that neutralized infectivity of 200 50% tissue culture infective dose of SARS-CoV. ^c^Immunofluorescent antibody test was performed by using a serum dilution of 1:50, and the intensity of staining in SARS-CoV–infected cells, but not in mock infected cells, was indicated by the sign +, with +++ representing the strongest signal observed. ^d^Western blot was conducted by using a serum dilution of 1:50, and the reactivity with the recombinant N protein expressed was indicated by the sign +, with ++++ representing the strongest signal observed.

**Figure:**
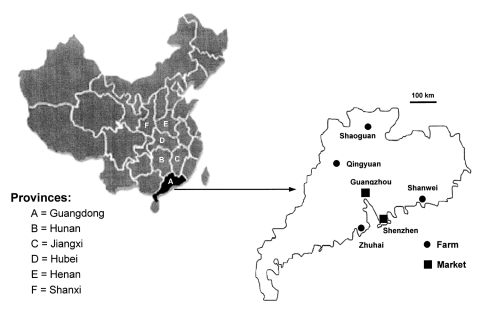
Geographic distribution of the farms and market examined in this study. The diagram on the left identifies the six provinces relevant to this study. The diagram on the right is an enlarged map of Guangdong Province showing the locations of the four farms and the capital city Guangzhou, where the live animal market was located. Also shown is Shenzhen, where civets from live animal markets were tested by Guan et al. in May 2003 (*5*).

Intestinal tissues collected from the 56 civets were tested by RT-PCR using N-gene primers; none of the samples were positive. Negative results were confirmed by RT-PCR with M- and S-gene primers. Therefore, virus isolation from these tissues was abandoned. The other 47 serum samples taken in June 2003 from Henan and Hunan provinces were negative by VNT or IFA (Table). Western blot was not performed on this group of serum samples.

## Discussion

While civet selection was derived from a convenience sample and limited because of time constraints imposed by the slaughter campaign, this study showed a marked difference in SARS-CoV antibody prevalence between animals from the market and those selected from the farms. Animals selected from one market in Guangzhou in January 2004 had a much higher prevalence of SARS-CoV antibodies than those selected from farms in the same period or from farms in two other provinces in June 2003. These results raise the possibility that civets, rather than being the natural animal reservoir of SARS-CoV, are infected mainly in markets or during other trade-related activities. Our results suggest that mass slaughter of civets on farms might not be necessary to control SARS-CoV spread. A more effective approach might be to implement testing in live animal markets and farms for susceptible animals and to apply quarantine regulation and targeted slaughter for markets or farms with infected animals.

While Guan et al. (*4*) were able to detect SARS-CoV infection by RT-PCR in six out of six palm civets collected in one particular live animal retail market in Shenzhen in May 2003, a similar study conducted by us in the same period yielded different results. In our study, we collected civets from Xinyuan Live Animal Market in Guangzhou (n = 7), the Guangdong Centre for Rescue and Care of Wildlife Animals, also located in Guangzhou (n = 9), and a civet farm in neighboring Jiangxi Province (n = 15). While 2 civets from the market and 2 from the center were positive for SARS-CoV by RT-PCR, all 15 farmed animals from Jiangxi had negative results (C. Tu et al., unpub. data).

Results of these studies and those from our current study are similar. We observed a high percentage of infected civets in one particular market at a specific time. However, no indication of civet infection was seen on most farms during the same period. These results support the hypothesis that civets are highly susceptible to SARS-CoV, perhaps especially when they are stressed, and that most infections occurred in the market.

We observed a number of practices during our study. First, most animal traders deal with multiple species. Second, housing different animals in close proximity is common. Third, although civets are in high demand in Guangdong Province, they are expensive, so a batch of animals may remain in a storehouse for weeks. All of these factors facilitate interspecies transmission, which would be followed by rapid transmission among the civet population. Finally, civet farming and trading has been in practice in China for >10 years, but SARS has not been observed in workers until recently, which points to a recent introduction of SARS-CoV in the civet population in markets.

In a study conducted by the Guangdong Province Centre for Disease Control and Prevention and the World Health Organization (*3*), epidemiologic data were analyzed from 1,454 clinically confirmed SARS cases (and 55 deaths) from November 2002 to April 30, 2003. One important observation from this study was that patients who became ill early in the epidemic were more likely than those who became ill later to report living near a produce market but not near a farm, which supports the notion that no widespread SARS-CoV infection occurred among farmed animals.

In the market study conducted by Guan et al. (*4*), all of the civets collected were positive for SARS-CoV. These animals were collected in the same market at the same time, but they originated in different regions of southern China; consequently, most, if not all, of these animals were likely infected in the market. In addition, SARS-CoV infection was also observed in at least one raccoon dog (*Nyctereutes procyonoides*) and one Chinese ferret-badger (*Melogale moschata*) from the same market at the same time, which demonstrates possible interspecies SARS-CoV transmission during trading. Sequence analysis of the S genes showed that one civet isolate (SZ16) was more closely related to the raccoon dog isolate (SZ13) than the other two civet isolates (SZ1 and SZ3), which further supports interspecies transmission in the market (*4*). Since that study, several experimental infection studies have shown most mammalian species tested to be susceptible to SARS-CoV infection (*5**–**9*), and animal-to-animal transmission can occur under experimental conditions as well (*6*). Caution should be taken in determining the origin of SARS-CoV; data collected from markets where a wide variety of species are housed in close proximity may be unreliable.

Out of the four farms in Guangdong Province, four animals from one farm in Shanwei had low levels of neutralizing antibodies to SARS-CoV, and two of the four samples did not react in IFA or Western blot. This farm in Shanwei is unique in that they farmed civets not for meat, but for the pet market in Southeast Asia. Most of their animals were obtained from various markets at various times from 2002 to 2003. These animals had possibly been exposed to SARS-CoV before arriving on the farm, and they still had low levels of convalescent antibodies in January 2004.

To assess the specificity of the serologic tests used in our study, we tested for cross-reactivity of SARS-CoV to four known coronaviruses from group 1 (porcine epidemic diarrhea virus and transmissible gastroenteritis virus), group 2 (porcine hemagglutinating encephalomyelitis virus), and group 3 (infectious bronchitis virus) and found no cross-reactivity (data not shown). We cannot rule out the possibility that an unknown coronavirus can infect civets, which may give low levels of cross-reactivity in the assays used in this study. However, such cross-reactive antibodies are not likely to positively react in all three of the assays used in this study.

The most basic limitation of our study was the nonrandom sampling, which limits the generalization of our results. However, this study is a first step in investigating the role of civets in transmitting SARS-CoV. Much remains to be done, including studies on the prevalence of infection with SARS-CoV and related coronaviruses that use more robust methods to sample susceptible animals in markets, farms, and the wild. Improved serologic tests should be developed that can detect SARS-CoV–specific antibodies from different animal species, without relying on live SARS-CoV. Other issues that remain to be resolved include the rate of new infections in susceptible animal species, the characteristics of the animals that become infected, and the nature of the exposures that lead to interspecies transmission.
